# Enforced C-Src Activation Causes Compartmental Dysregulation of PI3K and PTEN Molecules in Lipid Rafts of Tongue Squamous Carcinoma Cells by Attenuating Rac1-Akt-GLUT-1-Mediated Sphingolipid Synthesis

**DOI:** 10.3390/ijms21165812

**Published:** 2020-08-13

**Authors:** Chien-Wei Wu, Shyang-Guang Wang, Ching-Hsiao Lee, Wen-Ling Chan, Meng-Liang Lin, Shih-Shun Chen

**Affiliations:** 1 Division of Laboratory, Armed Force Taichung General Hospital, Taichung 411228, Taiwan; chien-wei@803.org.tw; 2 Department of Medical Laboratory Science and Biotechnology, Central Taiwan University of Science and Technology, Taichung 406053, Taiwan; sgwang@ctust.edu.tw; 3 Department of Medical Technology, Jen-The Junior College of Medicine, Nursing and Management, Miaoli 356006, Taiwan; heecs@ms38.hinet.net; 4 Department of Bioinformatics and Medical Enginerring, Asia University, Taichung 41354, Taiwan; wlchan@asia.edu.tw; 5 Department of Medical Laboratory Science and Biotechnology, China Medical University, Taichung 404394, Taiwan

**Keywords:** c-Src, casein kinase 2, cell invasion, gallic acid, GLUT-1, lipid raft, sphingolipid, PTEN, PI3K-Rac1-Akt, tongue squamous carcinoma

## Abstract

Pharmacologic intervention to affect the membrane lipid homeostasis of lipid rafts is a potent therapeutic strategy for cancer. Here we showed that gallic acid (GA) caused the complex formation of inactive Ras-related C3 botulinum toxin substrate 1 (Rac1)-phospho (p)-casein kinase 2 α (CK2α) (Tyr 255) in human tongue squamous carcinoma (TSC) cells, which disturbed the lipid raft membrane-targeting of phosphatidylinositol 3-kinase (PI3K)-Rac1-protein kinase B (Akt) signal molecules by inducing the association of p110α-free p85α with unphosphorylated phosphatase tensin homolog deleted on chromosome 10 (PTEN) in lipid rafts. The effects on induction of inactive Rac1-p-CK2α (Tyr 255) complex formation and attenuation of p-Akt (Ser 473), GTP-Rac1, glucose transporter-1 (GLUT-1) lipid raft membrane-targeting, and cell invasive activity by GA were counteracted either by CK2α short hairpin RNA or cellular-Src (c-Src) inhibitor PP1. PP1 treatment, GLUT-1 or constitutively active Rac1 ectopic-expression blocked GA-induced decreases in cellular glucose, sphingolipid and cholesterol of lipid raft membranes, p85α-p110α-GTP-Rac1 complexes, glucosylceramide synthase activity and increase in ceramide and p110α-free p85α-PTEN complex levels of lipid raft membranes, which reversed the inhibition on matrix metalloproteinase (MMP)-2/-9-mediated cell invasion induced by GA. Using transient ectopic expression of nuclear factor-kappa B (NF-κB) p65, MMP-2/-9 promoter-driven luciferase, and NF-κB-dependent luciferase reporter genes and NF-κB specific inhibitors or Rac1 specific inhibitor NSC23766, we confirmed that an attenuation of Rac1 activity by GA confers inhibition of NF-κB-mediated MMP-2/-9 expression and cell invasion. In conclusion, GA-induced c-Src activation is a key inductive event for the formation of inactive Rac1-p-CK2α (Tyr 255) complexes, which disturbed lipid raft compartment of PI3K and PTEN molecules by impairing Akt-regulated GLUT-1-mediated sphingolipid synthesis, and finally resulting in inhibition of TSC cell invasion.

## 1. Introduction

Aberration in lipid metabolism is known to be an important event for increasing construction of the cholesterol and sphingolipid-enriched membrane microdomain (lipid rafts) in developing cancers [1]. Among the constitution of lipid rafts, sphingolipids contain hydrophobic saturated fatty acyl chains that specifically and tightly interact with cholesterol to create formation of liquid-ordered microdomains, which therefore provides a platform for signal transduction by membrane receptor or participates in signaling events by recruiting signaling effectors to the membrane lipid rafts for the modulation of their activity [2]. Specifically, the signaling of the phosphatidylinositol 3-kinase (PI3K)-protein kinase B (Akt) pathway involved in cancer cell metabolism, survival, and invasion is modulated by lipid rafts [3,4]. The Ras-related C3 botulinum toxin substrate 1 (Rac1) can be selectively compartmentalized within the lipid rafts to regulate the oncogenic signaling of the PI3K-Akt axis [3,5,6]. The lipid raft association of Rac1 induces generation of the activated form of guanosine 5′-triphosphate-bound-Rac1 (GTP-Rac1), which is triggered by activated PI3K-induced production of phosphatidylinositol-3,4,5-trisphosphate (PIP_3_) at the plasma membrane [5]. The resultant GTP-Rac1 is recruited to the lipid rafts and binds to PI3K regulatory subunit p85α to enhance Akt activation [5]. The catalytical conversion of phosphatidylinositol 4,5-bisphosphate to PIP_3_ by PI3K is negatively modulated by the lipid raft-associated phosphatase and tensin homolog deleted on chromosome 10 (PTEN) [7]. The lipid phosphatase activity and membrane association of PTEN is enhanced by modulating the formation of p110-free p85α-unphosphorylated PTEN complexes [8]. Inactivation of casein kinase 2 (CK2) by CK2α small interfering RNA enhanced plasma membrane translocation of GTP-Rac1 [9]. The co-immunoprecipitation results revealed that CK2 was involved in suppression of Rac1 activation by direct association between catalytic CK2α subunit and inactive guanosine 5′-diphosphate-bound Rac1 (GDP-Rac1) [9]. Enhancement of CK2 catalytic activity is known to require phosphorylation of CK2α (Tyr 255) by the Src family protein kinase [10]. Although, many studies reveal the vital role of CK2 in cancer cell proliferation and survival [11], and its activation is shown to inhibit the transcriptional activity of stimulatory protein-1 (SP-1) by suppressing its DNA binding activity through phosphorylation of the carboxy-terminal domain of SP-1 [12]. In addition to gene regulation, CK2 also suppresses gelatinolytic activity by phosphorylating the matrix metalloproteinase-2 (MMP-2) to inhibit cancer cell invasion [13]. The upstream promoter sequences of *MMP-2* and *MMP-9* contain binding sites for the transcription factors, nuclear factor kappa B (NF-κB) and SP-1 [14,15]. Previous studies have demonstrated that NF-κB is a crucial mediator of *MMP-2* and *MMP-9* gene expression [16,17]. NF-κB has been considered as a potential regulator of cancer growth and invasion due to its function in the transcriptional regulation of antiapoptotic and *MMP* genes [18,19]. Gelatinolytic activities of MMP-2 and MMP-9 were associated with the invasiveness of tongue squamous carcinoma (TSC) cells [20]. These studies strongly indicate that Src-mediated CK may negatively regulate PI3K-Rac1-Akt-NF-κB signaling to modulate invasion of TSC cells.

Akt activation causes metabolic reprogramming of cancer cells by coordinating the glycolytic and sphingolipid metabolism through regulation of glucose uptake and metabolic enzyme activities or modulation of vesicle trafficking [21]. An elevated Akt activity involving in the high rate of glucose uptake to increase aerobic glycolytic capacity of cancer cells is achieved through directing of glucose transporter-1 (GLUT-1) to the cell surface [22,23,24]. Treatment with Akt-specific inhibitor (MK-2206) caused degradation of GLUT-1 in sustained Akt activation of breast cancer cells [25]. The connection between glucose metabolism and sphingolipid production is evidenced that reduction in glycosphingolipid levels by inhibition of glucosylceramide synthase leads to increase of glucose uptake and glycolytic metabolism in human leukemia HL-60 cells [26]. Furthermore, increased glucose uptake was found to increase the synthesis of glycosphingolipid [27]. It is proposed that the increased uptake and metabolism of glucose via Akt-stimulated lipid raft membrane targeting of GLUT-1 is a compensatory mechanism to rewire sphingolipid synthesis to reach homeostasis of membrane lipids during the carcinogenic process.

Gallic acid (3,4,5-trihydroxybenzoic acid, GA) is a naturally-occurring phenolic compound that exists in the seeds, fruits, and leaves of plants, such as grapes, berries, and tea [28,29]. This compound has been shown to display anti-invasive activity in human bladder cancer and melanoma cells by suppressing the PI3K-Akt-MMP-2 pathway [30]. Reduction in level of a crucial fatty acid synthase (FASN) by GA during de novo lipid synthesis was associated with inhibition of the invasive activity of human bladder cancer cells [30]. Elevated FASN activity is linked to improving invasive potential of cancer cells, which has been shown to upregulate synthesis of sphingolipids by increasing lipid biosynthesis [31]. Prior studies had demonstrated that GA-induced growth suppression of TSC cells was correlated to an increase of CK2 activity [32]. Hence, these observations motivate us to investigate the physiological role of lipid raft membrane-associated PI3K-Rac1-Akt effector molecules in modulating the GLUT-1-mediated glucose and lipid metabolism of the invasive potential of TSC cells, and to determine the molecular mechanism about how GA-induced CK2 activation affecting cell invasion.

## 2. Results

### 2.1. GA Inhibits TSC Cell Invasion by Downregulating MMP-2 and -9 Expression

**Figure 1:**
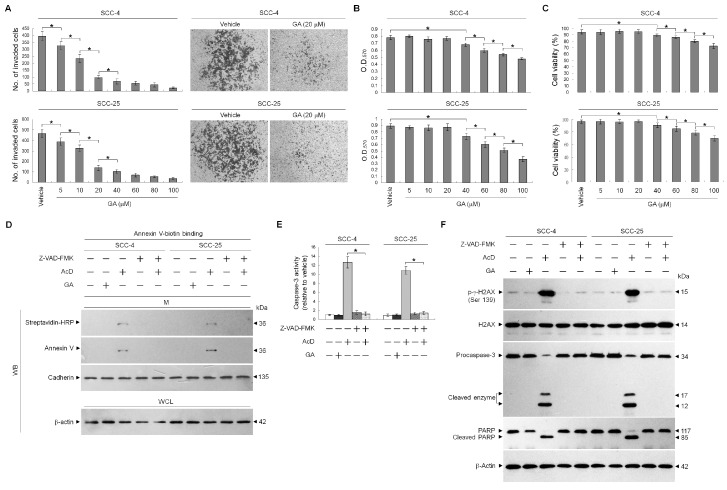
Gallic acid (GA) inhibits the invasive activity of human tongue squamous carcinoma (TSC) cells. (**A**–**C**) The effect of GA on TSC cell invasion. TSC cells were treated with vehicle (−) or the indicated concentrations of GA for 36 h. The number of invaded cells was assessed using the Matrigel invasion assay. Cell growth and viability were determined by the 3-(4,5-dimethylthiazol-2-yl)-2,5-diphenyltetrazolium bromide (MTT) assay and flow cytometric analysis of propidium iodide (PI) uptake, respectively. The values are presented as the means ± standard error (S.E.M.) of three independent experiments. * *p* < 0.05: significantly different from vehicle (−) or GA-treated cells. (**D**,**E**) The effect of GA on the induction of cancer cell apoptosis. After a 36 h treatment with vehicle, GA (20 μM), actinomycin D (ActD) (10 μM), or ActD and Ac-DEVD-CMK (8 μM), the caspase-3 activity was determined using flow cytometry. The values are presented as the S.E.M. of three independent experiments. * *p* < 0.05: significantly different from ActD-treated cells. Annexin V-biotinylated vehicle- or GA-treated cells were fractionated by subcellular fractionation centrifugation to isolate the plasma membrane (M) fraction. The level of the indicated proteins in the lysates of vehicle- or GA-treated whole cell (WC) and M fraction were determined by Western blot analysis using streptavidin-HRP and specific antibody to Annexin V. Antibody against cadherin was used as internal controls for the plasma membrane. β-Actin was used as an internal control for sample loading. (**F**) The levels of phosphorylated histone H2A.X (p-γ-H2AX), H2AX, poly (ADP-ribose) polymerase (PARP), and caspase-3 in the WCL were determined by Western blot analysis with specific antibodies. β-Actin was used as an internal control for sample loading.

In order to explore whether GA possess anti-invasive effect, an invasion assay was used to quantify cell invasion in a matrigel-coated chamber. Results from Figure 1A–C showed that GA applied at non-toxic concentrations (5–20 μM) decreased the invasive ability of the human TSC SCC-4 and SCC-25 cells in a dose-dependent manner. To verify that the decreased invasiveness was caused by the non-cytotoxic suppression of GA, rather than caspase-3 activation or apoptosis induction, caspase-3 activity and apoptotic markers were quantified by flow cytometry and determined by Western blot, while a broad spectrum caspase inhibitor Z-VAD-FMK was used. Annexin V-binding, caspase-3 activity, cleaved form of caspase-3 and PARP, and DNA double-strand break marker phosphorylated histone H2A.X (Ser 139) (p-γ-H2AX (Ser 139)) were not induced by 20 μM GA treatment, which displayed a similar phenomenon in cells treated with vehicle. Exposure of cells to the transcriptional inhibitor actinomycin D (ActD) resulted in induction of Annexin V-binding, caspase-3 activity and p-γ-H2AX (Ser 139) as well as proteolytic cleavage of inactive procaspase-3 and PARP to activated enzymes. The reaction was completely inhibited by the Z-VAD-FMK (Figure 1D–F), indicating that GA did inhibit invasiveness of TSC cells instead of causing apoptosis. Thus, a non-cytotoxic concentration of 20 μM was used to treat cells in all the following experiments. These results indicate that inhibition of TSC cell invasion by GS occurs independent of apoptosis induction.

**Figure 2:**
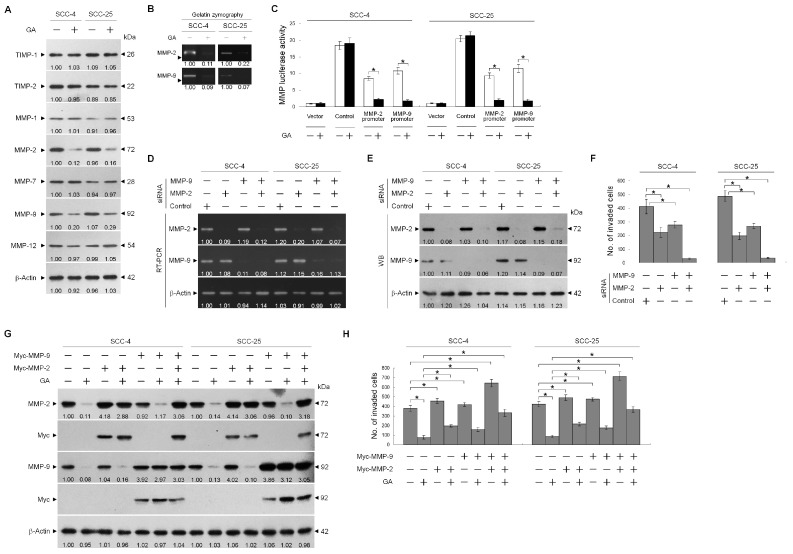
Suppression of tongue squamous carcinoma (TSC) cell invasiveness by gallic acid (GA) involves the downregulation of matrix metalloproteinase-2 (MMP-2) and MMP-9. (**A**) The effect of GA on MMP-2 and MMP-9 expression. The levels of tissue inhibitor of matrix metalloproteinases-1 (TIMP-1), TIMP-2, MMP-1, -2, -7, -9, and -12 in TSC cell lysates that were prepared after 36 h of treatment with vehicle (−) or GA (20 μM) were analyzed using specific antibodies. (**B**) At 36 h, the medium from vehicle- or GA (20 μM)-treated TSC cells was collected for analysis of the proteolytic activities of MMP-2 and MMP-9 by gel zymography. (**C**) The effect of GA on MMP-2 and MMP-9 promoter activities. TSC cells were co-transfected with the MMP-2 promoter and pCH110 plasmids or with the MMP-9 promoter and pCH110 plasmids for 12 h, followed by vehicle or GA (20 μM) for an additional 36 h. The MMP-2 and MMP-9 promoter activities in the cell lysates were determined using the luciferase reporter assay. The values are presented as the means ± standard error (S.E.M.) of three independent experiments. * *p* < 0.05: significantly different from the vehicle-treated, MMP-2 promoter plasmid-transfected, or MMP-P promoter plasmid-transfected cells. (**D**–**F**) Involvement of MMP-2 and MMP-9 in the invasiveness of TSC cells. TSC cells were transfected with the indicated small interfering RNAs (siRNAs) for 12 h followed by treatment with vehicle or GA (20 μM) for an additional 36 h. The levels of MMP-2 and MMP-9 transcripts and proteins in the siRNA-transfected cell lysates were determined by RT-PCR with specific primers and Western blot analysis using specific antibodies, respectively. The values above the figures represent relative density of the bands normalized to β-Actin. The cell invasion activity was determined using the Matrigel invasion assay. * *p* < 0.05: significantly different from the vehicle-treated, control siRNA-transfected cells. The values are presented as the S.E.M. of three independent experiments. (**G**,**H**) The effect of the ectopic expression of the Myc-MMP-2 and Myc-MMP-9 on the suppression of TSC cell invasion by GA. At 1 h after transfection with empty vector, Myc-MMP-2, Myc-MMP-9, or Myc-MMP-2 plus Myc-MMP-9, cells were treated with vehicle (−) or GA (20 μM) for 36 h. The levels of the indicated proteins in the whole cell lysates of transfected cells were determined by Western blot analysis with specific antibodies. The values above the figures represent relative density of the bands normalized to β-Actin. The cell invasion activity was determined using the Matrigel invasion assay. * *p* < 0.05: significantly different from the empty vector-transfected vehicle-treated cells or empty vector-transfected GA-treated cells. The values are presented as the S.E.M. of three independent experiments.

To investigate how GA regulated TSC cell invasion, the effects of GA on the expression of MMPs and anti-invasive tissue inhibitor of matrix metalloproteinases (TIMPs) were assessed. Western blot analysis showed that the treatment of TSC cells with GA decreased the levels of MMP-2 and -9 in cell lysates, although the levels of MMP-1, -7, and -12 remained the same. TIMP-1 and -2 expression levels were not increased in GA-treated cells (Figure 2A), and the enzymatic activities of MMP-2 and -9 were shown to be decreased in the culture media of GA-treated cells using gelatin zymography (Figure 2B). To determine whether GA reduced MMP-2 and -9 expression at the transcriptional level, we evaluated MMP promoter activity by transfecting TSC cells with a MMP-2 or -9 promoter-luciferase reporter constructs and then treated with GA. Figure 2C showed that the levels of both MMP-2 and -9 promoter activities were reduced by GA treatment. The invasion of TSC cells was significantly attenuated by MMP-2 or MMP-9 siRNA but not by control siRNA. Co-transfection of TSC cells with MMP-2 and MMP-9 siRNAs almost completely blocked cell invasion, confirming the results that both MMP-2 and MMP-9 are required for TSC cell invasion (Figure 2F). RT-PCR and Western blotting were used to validate knockdown of MMP-2 or MMP-9 expression by specific siRNA (Figure 2D,E). To investigate whether GA-induced inhibition of TSC cell invasion was associated with reduced expression of MMP-2 and MMP-9, we examined the effect of the ectopic expression of the Myc-MMP-2 and Myc-MMP-9 on GA-treated TSC cells. Expression of the Myc-tagged MMP-2 and MMP-9 proteins was confirmed by Western blot using anti-Myc antibody (Figure 2G). Compared with the control vector-transfected cells, the transfection of Myc-MMP-2 or Myc-MMP-9 slightly increased the number of invasive cells; however, Myc-MMP-2 or Myc-MMP-9 expression alone was not sufficient to overcome the suppression of cell invasion by GA. Cells expressing both Myc-MMP-2 and Myc-MMP-9 efficiently enhanced the invasive ability of TSC cells and exhibited rescue of the inhibitory effect of GA on the suppression of cell invasion (Figure 2H). These results indicated that the inhibition of TSC cell invasion by GA was most likely to be related to the downregulated expression of MMP-2 and -9.

### 2.2. GA Decreased Lipid Raft Membrane Targeting of PI3K, Rac1, Akt, and GLUT-1 Molecules

**Figure 3:**
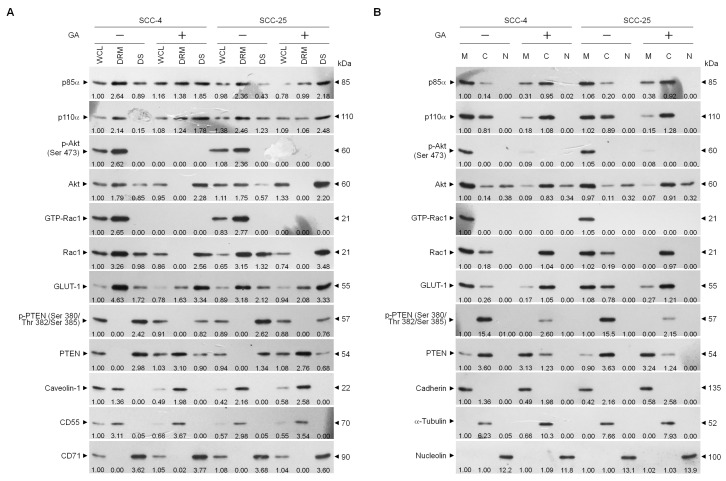
Gallic acid (GA) changes the lipid raft membrane localization of p85α, p110α, Ras-related C3 botulinum toxin substrate 1 (Rac1), protein kinase B (Akt), and phosphatase tensin homolog deleted on chromosome 10 (PTEN). (**A**,**B**) Tongue squamous carcinoma (TSC) cells were treated with vehicle (−) or GA (20 μM) for 36 h. Detergent-resistant membrane (DRM) and detergent-soluble (DS) fractions were prepared by flotation on a sucrose density gradient. Subcellular plasma membrane (M), cytosolic (C), and nuclear (N) fractions were separated by differential centrifugation. The levels of the indicated proteins in the lysates of vehicle- or GA-treated whole cell (WCL), DRM, DS, M, C, and N fractions were determined by Western blot analysis using specific antibodies. Antibodies against cadherin, α-tubulin, and nucleolin were used as internal controls for the plasma membrane, cytosol, and nucleus, respectively. β-Actin was used as an internal control for sample loading. The values above the figures represent relative density of the bands normalized to β-Actin.

Given that the spatial compartmentalization of signaling components in the lipid raft membranes provides a signaling hubs in cancer cell invasion [4], subsequent studies were performed to determine whether inhibition of TSC cell invasion by GA could result in a dysregulation of the lipid raft membrane targeting of PI3K-Akt signaling-regulated molecules. Detergent-resistant membranes (DRMs) were biochemically referred as lipid rafts, which were isolated by sucrose density gradient fractionation [33]. Western blots indicated that p85α, p110α, phosphorylated Akt (Ser 473), active GTP-bound Racl (GTP-Rac1), and GLUT-1 were mainly confined to the DRM fractions of vehicle-treated cells and associated with the lipid raft markers caveolin-1 and CD55 but not with the non-lipid raft marker CD71. Immunoreactivity only against few of p85α, p110α, unphosphorylated Akt, inactive GTP-unbound Rac1 (Rac1), and GLUT-1 could be observed in the CD71-enriched detergent-soluble (DS) fractions. In contrast, the phosphatase-inactive form of phospho-PTEN (p-PTEN) (Ser 380/Thr 382/Ser 385) was only observed in the DS fractions of vehicle-treated cells, whereas the biologically active form of PTEN (unphosphorylated PTEN; PTEN) was not found in the DRMs fractions. Exposure of cells to GA reduced the levels of p85α and p110α in the DRMs accompanied with increases of p85α and p110α in the DS fractions. However, GA showed enhanced lipid raft localization of unphosphorylated PTEN, in which unphosphorylated PTEN was increased in the DRM fractions with a concomitant decrease of phosphorylated PTEN (Ser 380/Thr 382/Ser 385) in the DS fractions. The levels of GTP-Rac1, p-Akt (Ser 473), and GLUT-1 were profoundly decreased in the DRM fractions after GA treatment, and that was related contingently to increased levels of inactive Rac1, unphosphorylated Akt, and GLUT-1 in the DS fractions (Figure 3A). We also examined the membrane localization of those effectors by a subcellular fractionation experiment and found that p85α, p110α, p-Akt (Ser 473), GTP-Rac1, and GLUT-1 were mostly defined to the membrane (M) fractions and associated with the plasma membrane marker cadherin [34]. There were also small amounts of p85α, p110α, inactive Rac1, and GLUT-1 in the cytosolic (C) fractions. Furthermore, we observed that most of the PTEN with phosphorylation on Ser 380, Thr 382, and Ser 385 residues distributed to the C fractions, with little unphosphorylated PTEN localizing to the M fractions. Treatment of GA led to an enhanced membrane targeting of unphosphorylated PTEN. GA treatment causes of decreased levels of p85α, p110α, and p-Akt (Ser 473) in the M fractions by increasing these proteins in the C fractions. Attenuated activation of Rac1 by GA correlated with the absence of GTP-Rac1 in the M fractions and increased level of inactive Rac1 in the C fractions. Only a small amount of GLUT-1 existed in the M fractions, while it was localized abundantly in the C fractions of GA-treated cells (Figure 3B). These results suggest that interferences of the lipid raft membrane localization and activation of p85α, p110α, Akt, Rac1, and GLUT-1 by GA, were associated with the lipid raft membrane targeting of unphosphorylated PTEN.

### 2.3. Perturbation of the Lipid Raft Membrane Targeting of PI3k-Rac1-Akt-Glut-1 Signaling Molecules and the Formation of P85α-Unphosphorylated PTEN Complexes Via the Inductive Effect of GA on the Formation of Inactive Rac1-P-Ck2α (Tyr 255) Complexes

**Figure 4:**
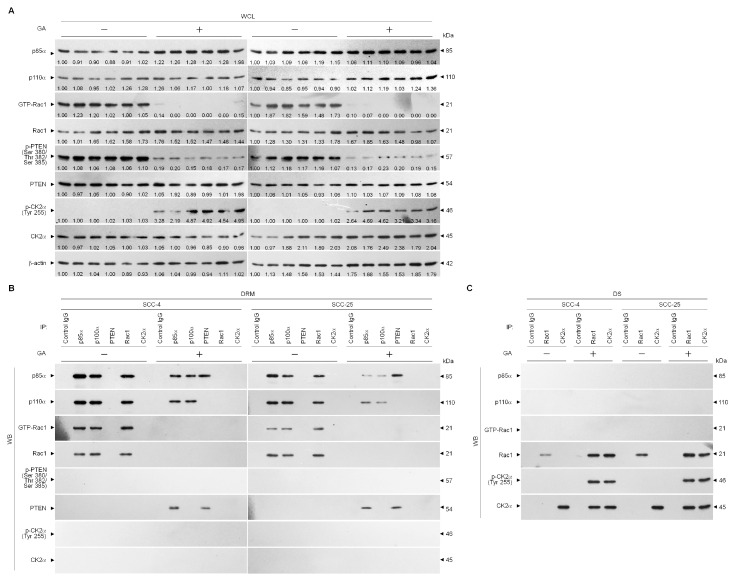
Gallic acid (GA) induces formation of inactive Ras-related C3 botulinum toxin substrate 1 (Rac1)–phospho (p)-casein kinase 2α (CK2α) (Tyr 255) and lipid raft-associated p85α–unphosphorylated phosphatase tensin homolog deleted on chromosome 10 (PTEN) complexes (**A**–**C**) Tongue squamous carcinoma (TSC) cells were treated with vehicle (−) or GA (20 μM) for 36 h. The levels of the indicated proteins in the lysates of vehicle- or GA-treated whole cell lysates (WCL) were determined by Western blot analysis using specific antibodies. The values above the figures represent relative density of the bands normalized to β-Actin. Coimmunoprecipitation of p85α, p110α, Rac1, PTEN, and CK2α was performed using the detergent-resistant membrane (DRM) and detergent-soluble (DS) fractions prepared from the cells treated as described above. The antibody used for coimmunoprecipitation is indicated at the top. The proteins from the immunoprecipitated complexes were detected using Western blotting with specific antibodies. Normal IgG was used as a control for antibody specificity.

Activation of the PI3K-Rac1-Akt pathway requires formation of cluster complexes in lipid raft membranes [35]. We next addressed whether GA treatment caused changes in the subcellular localization of key signaling components that may be due to the effect of physical interactions of p85α, p110α, Rac1, and PTEN in the lipid raft membranes. As shown in vehicle-treated cells of Figure 4B, we detected coimmunoprecipitation of the p85α, p110α, and GPT-Rac1 proteins but not PTEN from the DRM fractions using an antibody specific for p85α. The retro situation is observed with anti-p110α antibody which clearly coimmunoprecipitated with p85α and GPT-Rac1. When the cells were subjected to GA exposure, immunoprecipitates of anti-p85α with lysates of DRM fractions clearly included p85α:p110α:unphosphorylated PTEN, whereas immunoprecipitates obtained with anti-p110α antibody contained p85α:p110α. Reprecipitation with anti-PTEN antibodies, followed by anti-p85α, -p110α, -Rac1, PTEN, and -p-PTEN (Ser 380/Thr 382/Ser 385) immunoblot analysis was carried out to confirm that a complex with p110α-free p85α and unphosphorylated PTEN was detected in the DRM fractions of GA-treated cells. However, reduced coimmunoprecipitation of p85α and p110α was found, and failure to coimmunoprecipitate GPT-Rac1 was observed with p85α or p110α antibody in the DRM fractions of GA-treated cells. Control IgG was not able to coimmunoprecipitate any specific protein that interacted with the p85α, p110α, Rac1, or PTEN proteins, confirming the specificity of the p85α-p110α-GTP Rac1 or p85α-unphosphorylated PTEN complexes. These results indicated that GA induced an association between p110α-free p85α and unphosphorylated PTEN in the lipid raft membranes. Because abolished Rac1 activation was found to associate with physical interactions between inactive Rac1 and catalytic α subunit of casein kinase 2 (CK2) [9]. Induction of CK2 activity has also been addressed previously in GA-treated TSC cells [32], we investigated whether alterations in the interaction with CK2α contribute to inhibition of the lipid raft membrane association and activation of Rac1. Rac1 antibody was shown clearly to coimmunoprecipitate with p85α and p100α but not with phosphorylated CK2α (Tyr 255) in the DRM fractions of vehicle-treated cells (Figure 4B). p85α and p100α proteins were not coimmunoprecipitated with Rac1 from the DS factions of vehicle-treated cells as detected using Rac1 or CK2α antibody (Figure 4C). When cells were subjected to GA exposure, anti-Rac1 or -CK2α immunoprecipitations from the DRM fractions contained no p85α and p100α (Figure 4B); however, immunoprecipitated Rac1 formed complexes with p-CK2α (Tyr 255) in the DS fractions, and this phenomenon was further confirmed by co-immunoprecipitation with anti-CD2α antibody (Figure 4C). Based on these findings, we suggest that induction of the inactive Rac1-p-CK2α (Tyr 255) complex formation by GA plays an important event for initiating disruption of lipid raft membrane-associated PI3K-Rac1-Akt-GLUT-1-mediated signaling and causing the formation of p85α-unphosphorylated PTEN complexes.

### 2.4. Formation of Inactive Rac1-p-CK2α (Tyr 255) Complexes by GA-Induced C-Src Activation Attenuates Akt-Mediated Glut-1 Membrane Localization, and Suppresses MMP-2/-9-Mediated Cell Invasion

**Figure 5:**
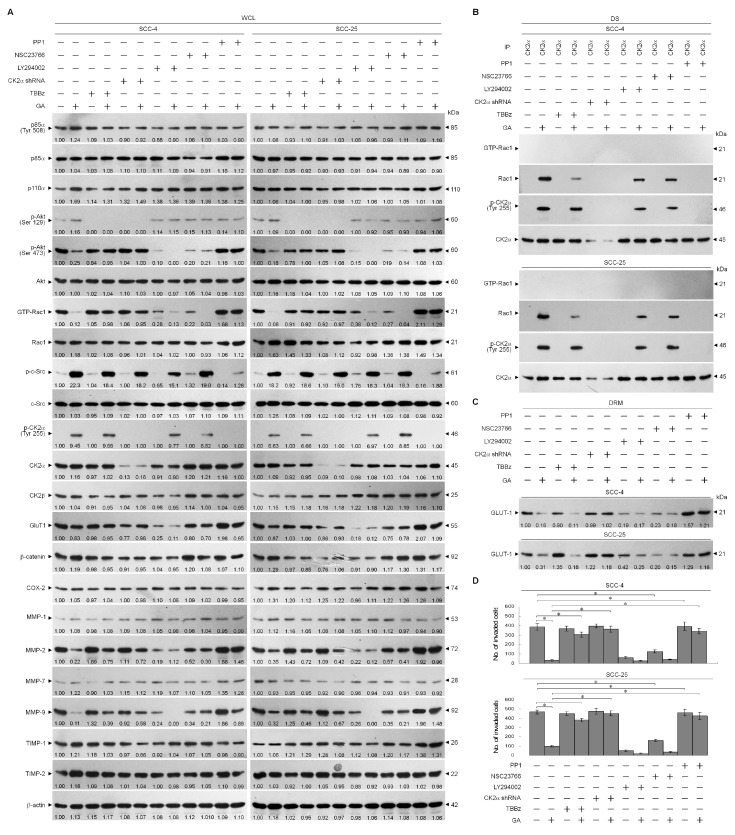
Formation of inactive Ras-related C3 botulinum toxin substrate 1 (Rac1)–phospho (p)-casein kinase 2α (CK2α) (Tyr 255) complexes by gallic acid (GA)-induced cellular-Src (c-Src) activation causes the attenuation of protein kinase B (Akt)-mediated glucose transporter-1 (GLUT-1) lipid raft membrane-targeting, thereby inhibits matrix metalloproteinase-2 (MMP-2)/-9-mediated cell invasion. At 12 h after transfection with the CK2α ShRNA or empty vector control, the cells were treated with vehicle (−), GA (20 μM), 4,5,6,7-tetrabromo-1H-benzimidazole (TBBz) (12 μM), GA (20 μM) plus TBBz (12 μM), 2-(4-morpho-linyl)-8-phenyl-4H-1-benzopyran-4-one (LY294002) (10 μM), GA (20 μM) plus LY294002 (10 μM), N6-[2-[[4-(diethylamino)-1-methylbutyl]amino]-6-methyl-4-pyrimidinyl]-2-methyl-4,6-quinolinediamine trihydrochloride (NSC23766) (50 μM), GA (20 μM) plus NSC23766 (50 μM), 1-(1,1-dimethylethyl)-3-(4-methylphenyl)-1H-pyrazolo [3,4-d]pyrimidin-4-amin (PP1) (6 μM), or GA (20 μM) plus PP1 (6 μM) for an additional 36 h. (**A**) The levels of the indicated proteins in the lysates of vehicle- or indicated compound-treated or CK2α short hairpin RNA (shRNA)-transfected cells were determined by Western blot analysis using specific antibodies. β-Actin was used as an internal control for sample loading. (**B**) The CK2α used for co-immunoprecipitation is indicated at the top of the figure. The anti-CK2α immunoprecipitations from the detergent-soluble (DS) fractions were immunoblotted with indicated antibodies. (**C**) GLUT-1 level in the detergent-resistant membrane (DRM) fractions was immunoblotted with GLUT-1 antibody. (**D**) The invaded cell numbers were assessed by the Matrigel invasion assays. The values are presented as the means ± standard error (S.E.M.) of three independent experiments. * *p* < 0.05: significantly different from vehicle (−) or GA-treated cells. The values above the figures represent relative density of the bands normalized to β-Actin.

Tyrosine kinase Src-dependent CK2α (Tyr 255) phosphorylation is important for the increased catalytic function of CK2 [10]. PI3K-Rac1-mediated sustained Akt activity was postulated to maintain GLUT-1 membrane trafficking and cell invasion [5,22,23]. We further investigated whether induced interaction of p-CK2α (Tyr 255) with inactive Rac1 by GA was involved in an activity of Src or PI3K. The catalytic α subunit of CK2, but not the regulatory β subunit, was readily phosphorylated by GA stimulation. Cellular-Src (c-Src) becomes heavily phosphorylated in response to GA treatment. The selectivity of PP1 for c-Src inhibition was evidenced by the loss of p-c-Src in cells treated with PP1 compared to vehicle alone. No changes were observed in phosphorylations of Akt (Ser 129), Akt (Ser 473), and CK2α (Tyr 255) as well as levels of p85α, p110α, Akt, GTP-Rac1, CK2β, GLUT-1, β-catenin, cyclooxygenase-2 (COX-2), MMP-1/-7, and TIMP-1/-2 in PP1-treated cells when MMP-2 and -9 levels were increased. Increased Tyr 255 phosphorylation of CK2α by GA was inhibited by PP1. PP1 addition also abolished the decrease of MMP-2/-9, the number of invaded cells, and the formation of inactive Rac1-CK2α complexes in cells stimulated with GA. Inhibition of Rac1 activity by specific inhibitor TBBz caused a slight increase in MMP-2/-9 levels and an decrease of p-Akt (Ser129), but not affecting the levels of p85α, p110α, p-Akt (Ser 473), GTP-Rac1, p-CK2α (Tyr 255), p-C-Src, CK2β, GLUT-1, β-catenin, COX-2, MMP-1/-7, TIMP-1/-2, and invaded cells. GA caused a decrease in the levels of phosphorylated Akt (Ser 473), GTP-Rac1, and invaded cells, and also downregulated the expression of MMP-2 and -9, which were reversed by TBBz; however, TBBz treatment did not block GA-induced phosphorylation of CK2α (Tyr 255). Suppression of CK2α expression by specific shRNA did not affect the levels of p-85α, p110α, p-Akt (Ser129), p-Akt (Ser 473), GTP-Rac1, p-CK2α (Tyr 255), p-C-Src, CK2β, GLUT-1, β-catenin, COX-2, MMP-1/-2/-7/-9, TIMP-1/-2, and invaded cells, but it did reduce p-Akt (Ser 129) level. Cells expressing CK2α shRNA in the presence of GA exhibited rescue effects on the suppression of Akt (Ser 473) phosphorylation, Rac1 activation, GLUT-1 lipid raft localization, MMP-2/-9, cell invasive activity, and the induction of inactive Rac1-CK2α complexes as well. The presence of PI3K specific inhibitor LY294002 alone resulted in decreased expression of GLUT-1, MMP-2, and -9 and suppressed cell invasion. Moreover, these protein and cell invasive levels decreased more strikingly under the combined treatment of GA (Figure 5A–D). The efficacy of LY294002 to block Akt activation was indicated by detecting a loss of phosphorylation of Akt (Ser 473). Blockade of Rac1 activation with a specific inhibitor NSC23768 was concomitant with decreased levels of p-Akt (Ser 473), MMP-2, and -9, and GA in combination with NSC23768 had a greater efficacy in reducing levels of these proteins (Figure 5A). These results indicated that GA induced c-Src-dependent phosphorylation of CK2α (Thr 255), leading to formation of inactive Rac1-p-CK2α (Thr 255) complexes, caused the attenuation of PI3K-Akt-mediated GLUT-1 lipid raft membrane-targeting, thereby inhibited MMP-2/-9-mediated cell invasion.

### 2.5. Impairment of Rac1 Activation by GA-Induced c-Src Activity Attenuates Akt-Regulated GLUT-1-Mediated Membrane Lipid Homeostasis, Leading to Inhibition of Cell Invasion

**Figure 6:**
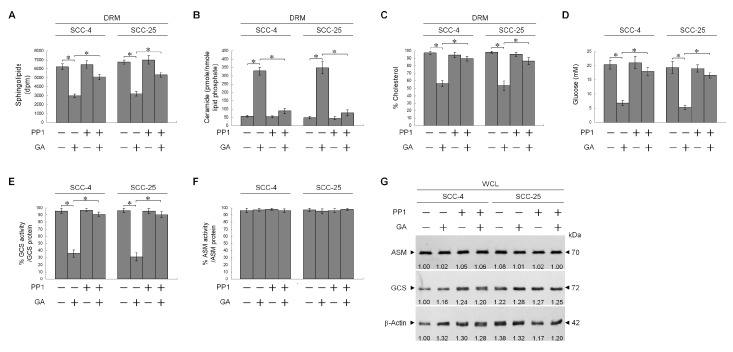
Gallic acid (GA)-induced cellular-Src (c-Src) activity confers perturbation of the lipid homeostasis of lipid raft membranes by suppressing glucosylceramide synthase (GCS) activity. Tongue squamous carcinoma (TSC) cells were treated with either vehicle (−), GA (20 μM), 1-(1,1-dimethylethyl)-3-(4-methylphenyl)-1H-pyrazolo[3,4-d]pyrimidin-4-amin (PP1) (6 μM), or GA (20 μM) plus PP1 (6 μM) for 36 h. (**A**–**F**) Cell viability was determined by the flow cytometric analysis of PI uptake. The lipids were extracted from the detergent-resistant membrane (DRM) fractions, and cholesterol, sphingolipid, and ceramide were quantitated by Amplex Red Cholesterol Assay Kit and thin-layer chromatography, respectively. Ceramide concentrations were normalized to phospholipid phosphate. The value of cellular glucose was analyzed using glucose assay kit. Acid sphingomyelinase (ASM) activity was determined by Acidic Sphingomyelinase Assay Kit. GCS activity was measured by addition of NBD-ceramide via thin-layer chromatography (TLC) separation. The values are presented as the means ± standard error (S.E.M.) of three independent experiments. * *p* < 0.05: significantly different from vehicle (−) or GA-treated cells. (**G**) The levels of the indicated proteins in the cell lysates were determined by Western blot analysis with specific antibodies. β-Actin was used as an internal control for sample loading. The values above the figures represent relative density of the bands normalized to β-Actin.

**Figure 7:**
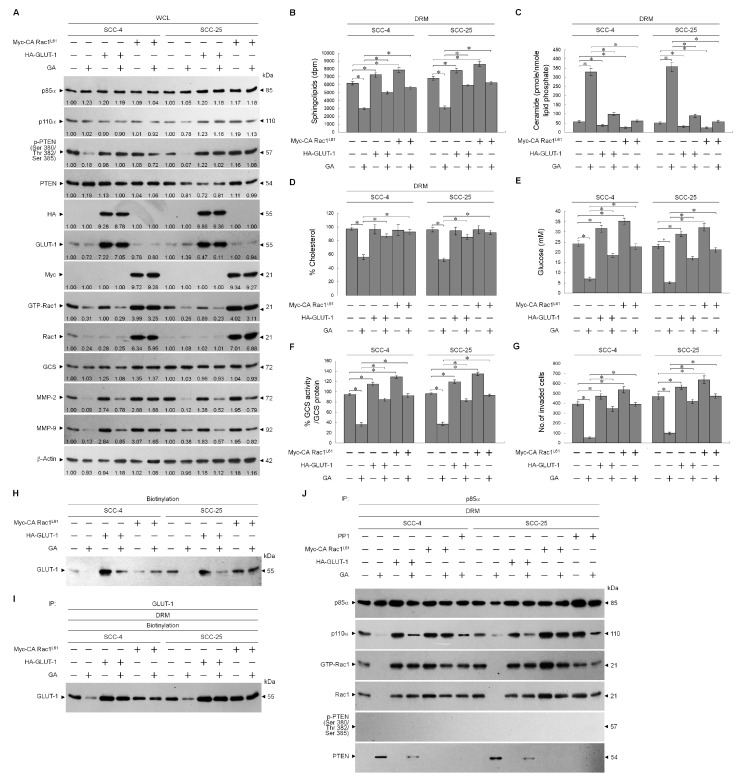
Ectopic expression of hemagglutinin-epitope-tagged glucose transporter-1 (HA-GLUT-1) or Myc-tagged constitutively active form of Ras-related C3 botulinum toxin substrate 1^L61^ (Myc-CA Rac1^L61^) overcomes gallic acid (GA)-induced formation of p85α–unphosphorylated phosphatase tensin homolog deleted on chromosome 10 (PTEN) and inhibition of GLUT-1-mediated lipid raft sphingolipid homeostasis and cell invasion. At 12 h after transfection with the HA-GLUT-1-, Myc-CA Rac1^L61^-expressing plasmids or empty vector control, the cells were treated with vehicle, GA (20 μM), or 1-(1,1-dimethylethyl)-3-(4-methylphenyl)-1H-pyrazolo[3,4-d]pyrimidin-4-amin (PP1) (6 μM) for an additional 36 h. (**A**) The levels of the indicated proteins in the cell lysates were determined by Western blot analysis with specific antibodies. β-Actin was used as an internal control for sample loading. The values above the figures represent relative density of the bands normalized to β-Actin. (**B**–**F**) The lipids were extracted from the DRM fractions, and cholesterol, sphingolipid, and ceramide were quantitated by Amplex Red Cholesterol Assay Kit and thin-layer chromatography, respectively. Ceramide concentrations were normalized to phospholipid phosphate. The value of cellular glucose was analyzed using glucose assay kit. Acid sphingomyelinase (ASM) activity was determined by Acidic Sphingomyelinase Assay Kit. Glucosylceramide synthase (GCS) activity was measured by addition of NBD-ceramide via thin-layer chromatography (TLC) separation. The values are presented as the means ± standard error (S.E.M.) of three independent experiments. * *p* < 0.05: significantly different from vehicle (−) or GA-treated cells. (**G**) The invaded cell numbers were assessed by the Matrigel invasion assays. The values are presented as the S.E.M. of three independent experiments. * *p* < 0.05: significantly different from vehicle (−) or GA-treated cells. (**H**) Biotinylated proteins were pulled down using streptavidin agarose beads. The biotin-streptavidin complexes were immunoblotted with GLUT-1 antibody. (**I**) The GLUT-1 antibody used for co-immunoprecipitation of cell surface-biotinylated protein from the detergent-resistant membrane (DRM) fractions is indicated at the top of the figure. (**J**) The p85α antibodies used for co-immunoprecipitation are indicated at the top of the figure. The proteins in the immunoprecipitated complexes from the DRM were analyzed by Western blot using specific antibodies.

PI3K-Rac1-Akt-mediated signaling is dependent upon the structure and function of lipid raft membrane [35]. Cholesterol and sphingolipid have shown to be critical for maintaining lipid raft structure [36,37]. Changes in composition of lipid rafts by increasing ceramide level perturb the integrity and dynamics of membranes, resulting in impaired cellular signaling and cell migration [38]. We examined whether structural composition of the lipid raft membranes was affected by GA. Caveolin-1- and CD55-enriched DRM fractions were analyzed for lipid components. Treatment with GA did reduce the sphingolipid and cholesterol contents of lipid raft membranes but increased ceramide generation (Figure 6A–C). No difference was observed on both acid sphingomyelinase (ASM) protein level and activity, when cells were treated with GA (Figure 6F,G). The results indicated that evaluation of ceramide level by GA was not due to an increase in ASM activity. Glucosylceramide synthase (GCS), converting ceramide to glucosylceramide, is an important enzyme for the synthesis of glycosphingolipids [39]. The level of GCS protein was not altered (Figure 6G), whereas this enzyme activity was reduced by GA (Figure 6E). The co-treatment of PP1 with GA returned the levels similar to those of sphingolipid, cholesterol, and ceramide in the lipid raft membranes of vehicle-treated cells (Figure 6A–C). GA-induced reduction of cellular glucose and GCS activity were restored to a similar level as cells treated with vehicle in combination of PP1 (Figure 6D,E). We next investigated if the reduction in sphingolipids and cholesterols and the elevation in ceramide synthesis by GA could be explained by impaired membrane targeting of GLUT-1 or Rac1 inactivation. HA-tagged GLUT-1 (HA-GLUT-1) or Myc-tagged constitutively active form of Rac1 (Myc-CA Rac1^L61^) expression construct was transfected into cells, and effects of their expression on GA-treated cells were examined. As shown by Western blot analysis, HA-GLUT-1 or Myc-CA Rac1^L61^ construct was able to achieve a higher level of GLUT-1 or Rac1 expression than that of the control vector alone. The expressions of HA-GLUT-1 and Myc-CA Rac1^L61^ proteins were verified by specific detection of HA- and Myc-tagged proteins (Figure 7A). An increased detection of the cell surface-associated GLUT-1 on the vehicle-treated ectopic HA-GLUT-1 expression was confirmed by Western blot analysis of streptavidin-agarose bead-bound protein from biotinylated cells (Figure 7H). Immunoprecipitation of cell surface-biotinylated proteins from the DRM fractions isolated with streptavidin-agarose beads using antibody specific for GLUT-1 showed an increase in the amount of GLUT-1 in the lipid raft membranes of vehicle-treated HA-GLUT-1-transfected cells, when compared with cells transfected with vehicle control (Figure 7I). Increased lipid raft membrane targeting of GLUT-1 by ectopic expression blocked the decrease of cellular glucose, sphingolipid, cholesterol, GCS activity levels and increase of ceramide induced by GA (Figure 7B–E), which simultaneously restored levels of p85α-p110α complexes and aborted p85α-unphosphorylated PTEN complex formation (Figure 7J). GA-induced inhibitions of MMP-2/-9 expression and cell invasion were overcome by ectopic expression of HA-GLUT-1 (Figure 7A,G). When Myc-CA Rac1^L61^ expressed in GA-treated cells, lipid raft membrane targeting of p85α-p110α complexes and GLUT-1 was restored, and the invasiveness and MMP-2/-9 expression properties were dramatically recovered (Figure 7A,G–J). GA-induced alterations in levels of sphingolipid, cholesterol, ceramide, and GCS activity were reversed by Myc-CA Rac1^L61^ overexpression (Figure 7B,D–F). These data suggest that invasion was suppressed in GA-treated cells by c-Src-dependent suppression of Rac1 activation. Therefore, cells losing Rac1 activity showed a decreased constitutive activity of Akt, which failed to maintain GLUT-1-mediated sphingolipid homeostasis of the lipid raft membranes and deregulated MMP-2/-9-mediated cell invasion.

### 2.6. GA-Induced Rac1 Inactivation Was Essential in Suppressing NF-κB-Mediated MMP-2/-9 Expression

**Figure 8:**
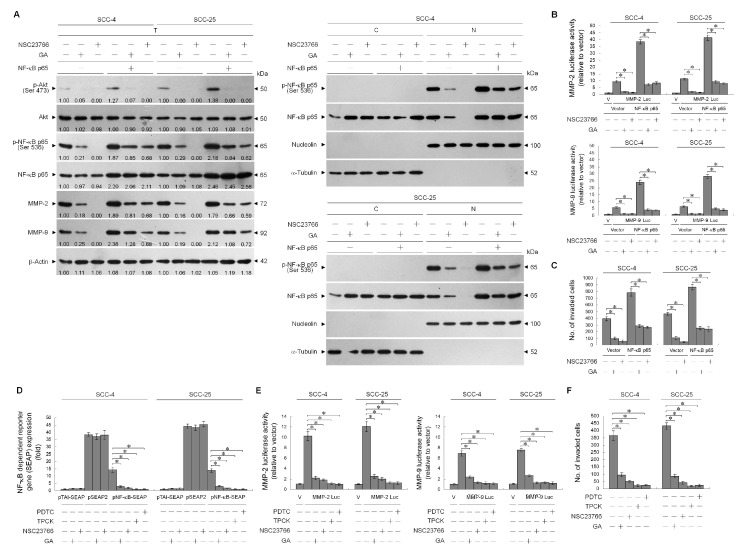
Ras-related C3 botulinum toxin substrate 1 (Rac1) inactivation is involved in gallic acid (GA)-induced inhibition of nuclear factor-kappa B (NF-κB)-mediated matrix metalloproteinase-2 (MMP-2)/-9 expression and cellular invasiveness. (**A**–**C**) At 12 h after transfection with the indicated plasmids, the cells were treated with the indicated compounds for an additional 36 h. The levels of the indicated proteins in the whole-cell, cytosolic, and nuclear extracts were then determined with specific antibodies. α-Tubulin and nucleolin were measured as internal controls for the cytosolic and nuclear fractions. The invaded cell numbers were assessed by Matrigel invasion assays. The values are presented as the means ± standard error (S.E.M.) of three independent experiments. * *p* < 0.05: significantly different from the vehicle-treated and vector-transfected cells or vehicle-treated and NF-κB p65-transfected cells. The values above the figures represent relative density of the bands normalized to β-Actin. (**D**–**F**) At 12 h after co-transfection with the indicated reporter plasmids, the cells were treated with the indicated compounds for an additional 36 h. The secreted alkaline phosphatase (SEAP), MMP-2 promoter, and invasive activities were then determined as described in Section 4. * *p* < 0.05: significantly different from the vehicle-treated, pNF-κB SEAP-transfected cells; the vehicle-treated, MMP-2 or MMP-9 promoter-transfected cells; or the vehicle-treated cells.

Rac1-regulated NF-κB transcriptional activity is required for MMP-2/-9-driven invasion of cancer cells [40]. PI3K-Akt signal-dependent Ser 536 phosphorylation of the NF-κB p65 subunit is implicated for the transactivation activity and nuclear translocation of NF-κB [41,42,43,44,45]. We sought to further investigate whether Rac1 inactivation was responsible for inhibition of cancer cell invasion by suppressing NF-κB-mediated MMP-2/-9 expression, by transient ectopic expression of NF-κB p65 in cells in the presence of GA or NSC23766. The Ser 536 phosphorylation and nuclear translocation of NF-κB p65 was inhibited by GA and NSC23766. NF-κB p65 ectopic expression resulted in marked increase in the level and nuclear localization of phosphorylated NF-κB p65 (Ser 536), and this induction raised MMP-2/-9 expression and cell invasive activity (Figure 8A,C). Furthermore, this effect of NF-κB p65 overexpression appeared to be promoter-driving specific, since luciferase expressions from the MMP-2 and -9 promoters that contained an upstream NF-κB-binding site were clearly induced by ectopic expression of NF-κB p65. The suppression of NF-κB p65 (Ser 536) phosphorylation and nuclear localization, MMP-2/-9 promoter activity and expression, and cell invasion by GA or NSC23766 could be overcome by the ectopic expression of NF-κB p65 (Figure 8A–C). We next examined whether GA affected transcriptions of NF-κB, MMP-2/-9 promoter-driven luciferase, and NF-κB-responsive secreted alkaline phosphatase (SEAP) activities in cells treated with GA, NSC23766, or NF-κB-specific inhibitor (TPCK or PDTC). Attenuation of the transcriptional regulation of NF-κB target *MMP-2* and *-9* genes was found to be dependent on the upstream inactivation of Rac1, as the inhibition of Rac1 activity by NSC23766 abrogated the NF-κB-driven SEAP and MMP-2/-9 promoter-driven luciferase activities. SEAP activity remained constant in the presence of NSC23766 or GA in cells transfected with the pSEAP control vector, which contains the *SEAP* gene under the control of the SV40 promoter. The results indicated that absence of non-specific regulation by Rac1 or GA was shown. Activities of NF-κB-driven SEAP, MMP-2/-9 promoter-driven luciferase, and cell invasion were attenuated by TPCK or PDTC treatment (Figure 8D–F). These results suggested that GA-induced inhibition of cell invasive activity was a result of the suppression of NF-κB-mediated MMP-2/-9 expression by Rac1 inactivation.

## 3. Discussion

**Figure 9:**
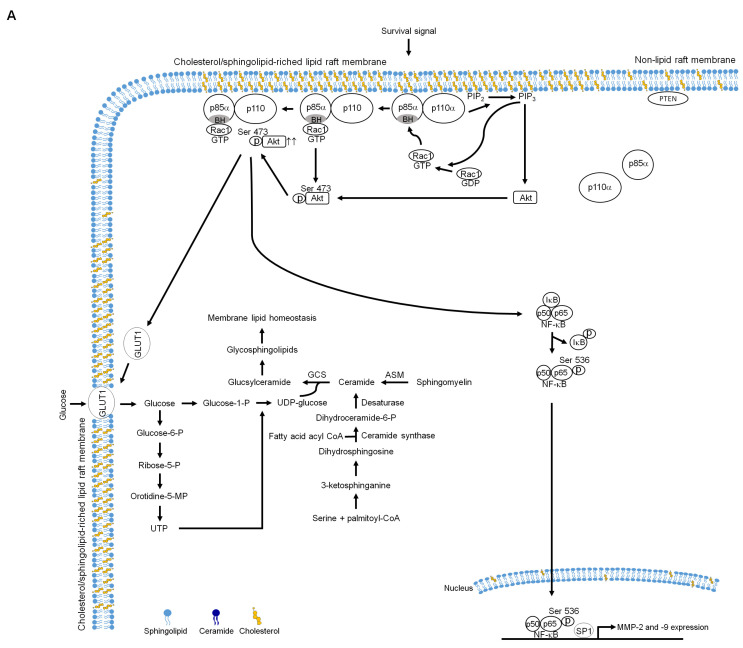

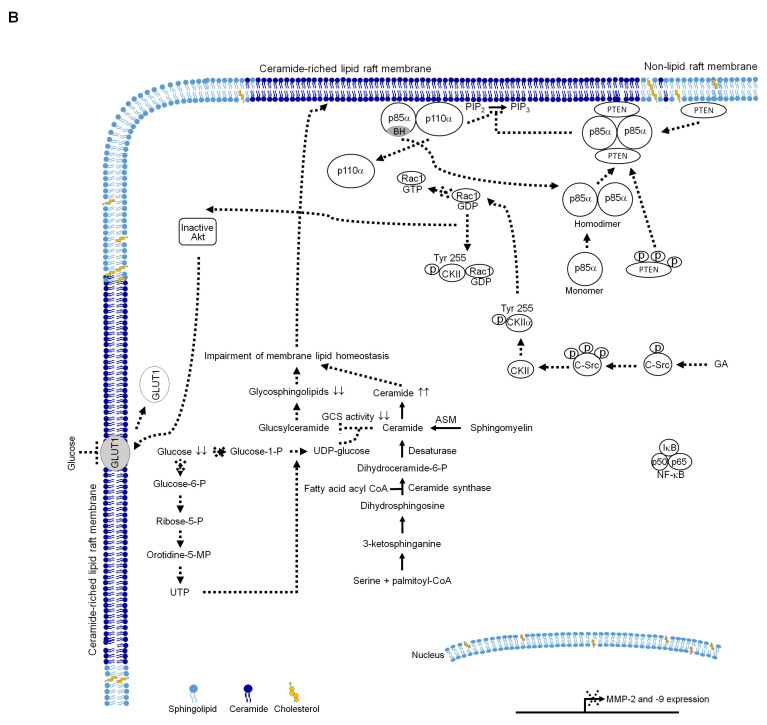
A molecular model for the induction of the inhibition of tongue squamous carcinoma (TSC) cell invasion by gallic acid (GA). (**A**) The selective interaction of clustered phosphatidylinositol 3-kinase (PI3K)-GTP-Ras-related C3 botulinum toxin substrate 1 (Rac1)-protein kinase B (Akt) signaling molecules in the lipid raft membranes constitutes a central element in the regulation of the lipid homeostasis of lipid raft membrane to promote cell invasive activity by enhancing lipid raft-associated glucose transporter-1 (GLUT-1)-mediated sphingolipid synthesis in response to survival signals. (**B**) Under the condition of cellular GA uptake, GA triggers cellular-Src (c-Src) activation to promote the Tyr 255 phosphorylation of casein kinase 2 α (CK2α), thus inducing the formation of inactive Rac1–p-CK2α (Tyr 255) complexes and thereby disturbing the lipid raft membrane localization and full activation of Akt. The resultant attenuated Akt failed to modulate the lipid raft membrane-stabilizing activity of GLUT-1, leading to the loss of GLUT-1-dictated sphingolipid synthesis and glucosylceramide synthase activity (GCS) activity, which in turn raises ceramide level to create the ceramide-rich lipid raft membranes. The generation of the ceramide-rich lipid raft membranes impairs compartment-specific localization of lipid raft-associated PI3K molecules, leading to the formation of p110α-free p85α-unphosphorylated phosphatase tensin homolog deleted on chromosome 10 (PTEN) complexes in the lipid raft membranes. The resultant lipid raft membrane-associated p85α–PTEN complexes can negatively regulate Akt activity by dephosphorylating the 3-position of phosphatidylinositol-3,4,5-trisphosphate (PIP_3_) to PIP_2_. Dysregulation of lipid raft-associated PI3K-Rac1-Akt signaling was accompanied by attenuation of nuclear factor-kappa B (NF-κB) regulated matrix metalloproteinase-2 (MMP-2)/-9-mediated cellular invasion.

Emerging evidence has shown that lipid metabolic reprogramming contributes to alter membrane dynamics and intracellular signal transduction pathways, which leads to chemotherapeutic resistance and anti-apoptotic action of cancer cells [46]. Changes in lipid metabolism elevates the levels of membrane sphingolipid and cholesterol that alter the membrane fluidity and protein dynamics of cancer cells to facilitate signaling protein recruitment, leading to create a higher presence of lipid raft platforms for cell survival [1,36]. Among the components of the plasma membranes, sphingolipids play a critical role for cancer cell survival and invasion by modulating the formation of a lipid raft-based sorting platform [2]. Lipid rafts serve as platforms for the recruitment and activation of Akt and Rac1 by PI3K and the sorting of specific lipids, and hubs for signal transduction [3,4,5]. Aberrant activation of the PI3K-Akt signal pathway was implicated in the downregulation of ceramide synthesis by triggering the generation of sphingomyelin to promote growth of glioma cells [47]. Elevated cellular ceramide by addition of ceramide analogs, C(2)- or C(6)-ceramide, is capable of attenuating Akt activation by reducing phosphorylation level of Akt (Ser 473) [48]. Induction of Akt (Ser 473) phosphorylation and kinase activity are dependent on its location in the lipid rafts [49]. It would be hypothesized that the PI3K-Rac1-Akt signal pathway acts as a decisive role in coordinating the lipid metabolism of cancer cells to modulate the lipid homeostasis of the lipid rafts. Therapeutic strategy design against PI3K-Rac1-Akt-modulated lipid metabolism of cancer cells is considered as a promising way to treat cancers [1,50]. Here we report that lipid rafts of GA-treated TSC cells with reduced levels of sphingolipid and cholesterol as well as an increased level of ceramide affect the organization of PI3K-Rac1-Akt signaling cascades formed from lipid raft membranes that impair cell invasion. Comprehensive examination of the light buoyant-density DRM lipid rafts and fractionation of light-membrane fractions revealed that GA altered distribution of several effectors in the lipid raft membranes, including the lipid raft membrane disassociation of p110α, Rac1, Akt, and GLUT-1 and the targeting of PTEN to the lipid raft membrane. Co-immunoprecipitation experiments performed in the DRM fraction lysates of GA-treated cells showed reduced level of p85α-p110α complexes and enhanced formation of p85α-unphosphorylated PTEN complexes in the lipid raft membranes, which were counter-regulated by PP1, GLUT-1, or CA Rac1 overexpression. In addition, there was observed recovery in the lipid raft membrane-targeting of p85α-p110α-GTP-Rac1 complexes and GLUT-1 and restored MMP-2/-9 expression level, cell invasive activity, and levels of sphingolipid and cholesterol in the lipid raft membranes. In view of these findings and the observed restoration of GLUT-1 lipid raft membrane-targeting, sphingolipid and cholesterol of lipid raft membranes, and cell invasive activity and attenuation of inactive Rac1-p-CK2α (Tyr 255) complex formation after silencing of CK2α by shRNA or inactivation of c-Src by PP1. It is logical to suggest that induction of phosphorylation-dependent activity of the c-Src by GA causes an occurrence of phosphorylated CK2α (Tyr 255) triggering inactive Rac1-p-CK2α (Tyr 255) interaction, thereby interfering with the activation of Rac1 and disrupting the recruitment of Akt into the lipid raft membranes to decrease Akt activation. The resultant attenuated Akt fails to modulate the lipid raft membrane-stabilizing activity of GLUT-1, leading to the loss of GLUT-1-dictated sphingolipid synthesis and GCS activity, which in turn raises ceramide level and affected compartment-specific localization of lipid raft-associated PI3K molecules inducing p110α-free p85α-unphosphorylated complexes in the lipid raft membranes. Dysregulation of lipid raft-associated PI3K-Rac1-Akt signaling was accompanied by attenuation of NF-κB regulated MMP-2/-9-mediated cellular invasion (Figure 9).

Uses of selective ATP-competitive CK2 inhibitor TBBz [51] and PP1 showed that the interaction between inactive Rac1 and p-CK2α (Tyr 255) was induced by GA in a c-Src-dependent CK2α (Tyr 255) phosphorylation- and CK2 activity-dependent manner as observed with the restoration of MMP-2/-9 expression and cell invasion by suppressing inactive Rac1-p-CK2α (Tyr 255) complex formation. Phosphorylation of Tyr 255 has been implicated in enhancing transactivation of CK2 for Akt Ser 129 phosphorylation-dependent β-catenin expression and cell survival [52,53]. Although this observation is inconsistent with increase of p-Akt (Ser 129) and β-catenin expression levels by GA treatment, GA blocks TSC cell invasiveness and p-Akt (Ser473) phosphorylation and suppresses the expression and promoter activity of MMP-2/-9 without significantly affecting cell viability and growth. As the study used human embryonic kidney HEK293 cells for functional assay, the physiological differences between the TSC and HEK293 cells of the reported findings need to be clarified further.

Although the fact that increased level or constitutive activation of c-Src was implied in the metastasis or carcinogenesis of colon, breast, lung, liver, pancreas, and prostate cancer cells [54,55,56], c-Src phosphorylation-dependent induction of hexokinase activity has been shown to stimulate the conversion of glucose to glucose-6-phosphate (G-6-P) associated with the carcinogenic and metastatic processes of cells [57]. Low level of cellular c-Src phosphorylation was found in the TSC cells. GA-caused increase of c-Src phosphorylation was accompanied by an induction of CK2α (Tyr 255) and enhanced its interaction with inactive Rac1 to attenuate Akt-regulated GLUT-1-mediated sphingolipid synthesis. This discrepancy in c-Src activity may be due to genetic changes and biochemical signaling alterations associated with reprogramming of energy metabolism. GA treatment did not induce the kinase activity of c-Src immunoprecipitates (data not shown). We do not exclude the possibility that additional unknown cellular factors or kinase may contribute to the GA-induced c-Src phosphorylation. Although the mechanism by which c-Src phosphorylation by GA is not known to date, there has been no evidence showing a link between enforced activation of c-Src by GA and attenuation of Akt-regulated GLUT-1-mediated sphingolipid synthesis to impair compartmental regulation of PI3K-Rac1-Akt signal molecules in human TSC cells.

The homeostasis of the biological properties of lipid raft membranes by facilitating synthesis of sphingolipids was tightly regulated by PI3K-Rac1-Akt-regulated GLUT-1-mediated glycolytic metabolism, as evidenced by decreased Akt (Ser 473) phosphorylation and Rac1 activation together with heightened level of lipid raft membrane ceramide, decreased sphingolipid, cholesterol, and GLUT-1 levels in the lipid raft membranes, loss of glucose and GCS activity, and reduced MMP-2/-9 expression and invasive activity in TSC cells treated with LY294002. The formation and lipid raft membrane association of the PTEN and p85α complexes induced by substitution of the cholesterol of the lipid rafts with ceramide confers attenuation of PI3K-Akt signaling, and this finding is consistent with previous studies [3,8,35,58]. The existence and role of Akt-regulated GLUT-1-mediated sphingolipid synthesis are providing new insights into the dynamic process occurring in the compartmental regulation of PI3K-Rac1-Akt signal molecules.

## 4. Materials and Methods

### 4.1. Cell Culture

The human tongue squamous carcinoma SCC-4 and SCC-25 cell lines were obtained from the Food Industry Research and Development Institute (Hsinchu, Taiwan). The cell lines were cultured routinely in Dulbecco’s modified Eagle’s medium (DMEM) supplemented with 5% fetal bovine serum (FBS) (both from Gibco BRL, Grand Island, NY, USA) and grown in 10-cm tissue culture dish at 37 °C in a humidified incubator containing 5% CO_2_.

### 4.2. Chemicals, Reagents, and Plasmids

Actinomycin D (ActD), N-acetyl-D-sphingosine (C2-ceramide), amitriptyline, Brij 98, crystal violet, 1,2-dioleoyl-sn-glycero-3-phosphocholine (DOPC), 2-(4-morpho-linyl)-8-phenyl-4H-1-benzopyran-4-one (LY294002), gallic acid (GA), glutathione agarose beads, NBD-C6-ceramide, propidium iodide (PI), N6-[2-[[4-(diethylamino)-1-methylbutyl]amino]-6-methyl-4-pyrimidinyl]-2-methyl-4,6-quinolinediamine trihydrochloride (NSC23766), pyrrolidine dithiocarbamate (PDTC), Tris-HCl, Triton X-100, 3-(4,5-dimethylthiazol-2-yl)-2,5-diphenyltetrazolium bromide (MTT), and N-*p*-tosyl-_L_-phenyl-alanine chloromethyl ketone (TPCK) were obtained from Sigma-Aldrich (St. Louis, MO, USA). NBD-C6-ceramide was dissolved in dH_2_O (0.5 mM) and stored at −20 °C. Methanol and potassium phosphate were purchased from Merck (Darmstadt, Germany). 4,5,6,7-tetrabromo-1H-benzimidazole (TBBz) and 1-(1,1-dimethylethyl)-3-(4-methylphenyl)-1H-pyrazolo[3,4-d]pyrimidin-4-amin (PP1) were purchased from Calbiochem (San Diego, CA, USA). GA was dissolved in and diluted with methanol and then stored at −20 °C as a 100 mM stock solution. Lipofectamine 2000 was obtained from Invitrogen (Carlsbad, CA, USA). FBS, trypsin-EDTA, and glutamine were obtained from Gibco BRL (Grand Island, NY, USA). DMEM, FBS, penicillin-streptomycin, trypsin-EDTA, and glutamine were obtained from Gibco BRL (Grand Island, NY, USA). Engelbreth-Holm-Swarm sarcoma tumor (EHS) extract Matrigel was obtained from BD Biosciences (Bedford, MA, USA). The caspase-3 activity assay kit was purchased from OncoImmunin (Gaithersburg, MD, USA). The caspase-3 inhibitor Ac-DEVD-CMK and N-acetyl-L-cysteine (NAC) were purchased from Calbiochem (San Diego, CA, USA). Centricon YM-100 was obtained from Millipore. Acid sphingomyelinase (ASM) Assay Kit (Fluorimetric) was obtained from Abcam (Cambridge, MA, USA). The Amplex Red cholesterol assay kit was purchased from Molecular Probe (Eugene, OR, USA). The MMP-2 and MMP-9 promoter luciferase reporter constructs were prepared as described previously [59]. The β-galactosidase expression vector and pCH110 were purchased from Amersham Pharmacia Biotech (Piscataway, NJ, USA). pCK2α shRNA, human influenza hemagglutinin (HA)-GLUT-1, and pCMV4-NF-κB p65 were obtained from Addgene (Cambridge, MA, USA). The luciferase assay kit was obtained from Promega (Madison, MI, USA). Full-length MMP-2 (NCBI ACCESSION NM_001127891 VERSION NM_001127891.2) and MMP-9 (NCBI ACCESSION NM_004994 VERSION NM_004994.3) cDNA sequences were generated by reverse-transcription PCR using total RNA from the SCC-4 cell line. PCR primers that include proper restriction enzyme sites were designed to be cloned into the pcDNA3.1/Myc vector. The cDNA sequence of individual clones was verified by automated DNA sequencing. Western blot luminol reagent was purchased from Santa Cruz Biotechnology. The primer sequences were as follows: MMP-2, 5′-AGCGAGTGGATGCCGCCTTTAA-3′ and 5′-CATTCCAGGCATCTGCGATGAG-3′; MMP-9, 5′-GCCACTACTGTGCCTTTGAGTC-3′ and 5′-CCCTCAGAGAATCGCCAGTACT-3′; β-actin, 5′-GCTTGACTCAGGATTTAAAAACTGGAACGG-3′ and 5′-TATTCAACTGGTCTCAAGTCAGTGTACAGG-3′.

### 4.3. Antibodies

Antibodies against MMP-1 and MMP-9 were purchased from Oncogene Research Product (Cambridge, MA, USA). Antibody against caspase-3 was obtained from Calbiochem (San Diego, CA, USA). Anti-Akt, -phospho (p)-Akt (Ser 473), p-Akt (Thr 34), and -NF-κB p65 antibodies were purchased from BD PharMingen. Anti-p-NF-κB (Ser 536) antibody was obtained from Cell Signaling Technologies (Boston, MA, USA). Antibodies directed against ASM and calnexin were obtained from Abcam (Cambridge, MA, USA). Antibodies against CD55, CD71, nucleolin, p110α, poly (ADP-ribose) polymerase (PARP), and Rac1 were purchased from Santa Cruz Biotechnology. Anti-pan-cadherin, anti-PTEN, and anti-p-PTEN (Ser 380/Thr 382/Ser 385) antibodies were obtained from Thermo Fisher Scientific (New York, NY, USA). Antibody against β-actin was obtained from Sigma-Aldrich. Peroxidase-conjugated anti-mouse, -goat, and -rabbit IgG secondary antibodies were purchased from Jackson ImmunoResearch Laboratory (West Grove, PA, USA).

### 4.4. Cell Viability Assay

Cell viability was assessed by fluorescence-activated cell sorting (FACS) analysis of cellular PI uptake [59]. Cells were seeded at 3 × 10^4^ cells/well in 24-well tissue culture plates. Cells were grown overnight to ≈60% confluence and treated with either DMSO as the vehicle control, or GA for 36 h. Serving as a vehicle control, methanol was diluted in culture medium to the same final concentration of methanol (0.1%; *v*/*v*) as GA. After incubation, treated cells were harvested and stained with PI solution (10 μg/mL) in phosphate-buffered saline (PBS). The samples were analyzed on a FACSCount flow cytometer (BD Biosciences, Franklin Lakes, NJ, USA). Cell Quest software (BD Biosciences, Franklin Lakes, NJ, USA) was used to analyze the results. PI-negative populations were defined as viable cells.

### 4.5. Determination of Caspase-3 Activity

Caspase-3 activity was measured using the PhiPhiLux G1D2 kit (OncoImmunin, College Park, MD, USA) according to the manufacturer’s protocols. Briefly, treated cells were incubated with PhiPhiLux fluorogenic Caspase substrate at 37 °C for 1 h and then analyzed using a FACSCount flow cytometer. The samples were assessed using a FACSCount flow cytometer, and the results were analyzed using CellQuest software.

### 4.6. RT-PCR

RNA was extracted using Trizol reagent (Invitrogen Life technologies, Carlsbad, CA, USA). First-strand cDNA was made using 1 μg of total RNA in the presence of Superscript II reverse transcriptase (Gibco/BRL) and random hexamers. cDNA was PCR-amplified using Vent DNA polymerase (NEB BioLabs) and specific primers corresponding directly to a region in the MMP-2 or MMP-9 gene. Samples were taken from the PCR mixture after 35 cycles of amplification, separated on a 1.5% agarose gel, and stained with ethidium bromide.

### 4.7. Density-Based Membrane Flotation Technique

Treated cells were washed twice in ice-cold PBS and scraped from dishes. Cells were then harvested by centrifugation, resuspended in 1 mL of hypotonic lysis buffer (10 mM Tris (pH 7.5), 10 mM KCl, 5 mM MgCl_2_) containing 0.5% Brij 98, incubated at 37 °C for 5 min, and ruptured by 20 passages through a 25-gauge hypodermic needle. Unbroken cells and nuclei were removed by centrifugation at 1000× *g* for 5 min in a microcentrifuge at 4 °C. The crude homogenates were maintained on ice for an additional 5 min, mixed with 3 mL of 72% sucrose, and overlaid with 4 mL of 55% sucrose and 1.5 mL of 10% sucrose; all of the sucrose solutions were dissolved in low-salt buffer (50 mM Tris-HCl (pH 7.5), 25 mM KCl, 5 mM MgCl_2_). The samples were centrifuged for 14 h in a Beckman SW41 rotor at 38,000 rpm and 4 °C. Fractions were collected from the top of the gradient in 1 mL increments and concentrated to approximately 100 μL by passage through a 50 kDa Centricon filter.

### 4.8. Western Blot and Co-Immunoprecipitation

Treated or transfected cells were separately lysed and subjected to Western blotting as described previously [3]. For co-immunoprecipitation, cellular extracts were immunoprecipitated with anti-p85α, -p110α, PTEN, Rac1, CK2α, and GLUT-1 antibodies, or with IgG as a normal control, and then incubated with protein A agarose beads as previously described [3]. After incubation at 4 °C for 2 h, the immune complexes were analyzed by 10% SDS-PAGE and immunoblotting with anti-p85α, anti-110α, anti-Rac1, anti-PTEN, anti-p-PTEN (Ser 380/Thr 382/Ser 385), anti-CK2α, anti-p-CK2α (Tyr 255), and anti-GLUT-1 antibodies. Densitometric measurements of the band in Western blot analysis were performed using computing densitometer and ImageQuant software (Molecular Dynamics, Sunnyvale, CA, USA).

### 4.9. Cell Surface Biotinylation

This assay was performed as previously described [59]. Briefly, treated cells were washed twice in ice-cold PBS and incubated with 0.5 mg/mL of EZ-Link Sulfo-NHS-SS-Biotin (Pierce, Rockford, IL, USA) for 30 min at 4 °C. Biotinylated cells were washed twice in ice-cold PBS and treated with 50 mM NH_4_Cl for 10 min at 4 °C to stop the biotinylation reaction. Avidin-agarose beads (Pierce, Rockford, IL, USA) were then added to the biotinylated cells, and the mixture was incubated with gentle rocking at 4 °C for 16 h. The beads were pelleted and washed three times with 500 μL of ice-cold PBS. Bound proteins were mixed with 1× SDS sample buffer and incubated for 5 min at 100 °C. The proteins were then separated by 10% SDS-PAGE and immunoblotted with antibody against GLUT-1.

### 4.10. Rac1 Activation Assay

Treated cells or caveolin-1 and CD55-riched DRM fractions prepared from treated cells were lysed by incubation with Rac1 lysis buffer (50 mM Tris-HCl (pH 7.4), 100 mM NaCl, 1 mM MgCl_2_, 20 mM β-glycerophosphate (pH 7.5), 1% NP-40, 10% glycerol, 10 mM NaF, 2 mM Na_3_VO_4_, 5 mM dithiothreitol, 0.5 mM phenylmethylsulfonyl fluoride, 1 μg/mL leupeptin, and 1 μg/mL pepstatin) for 15 min at 4 °C. The lysates were centrifuged at 14,000× *g* for 10 min in a microcentrifuge at 4 °C. The lysates and immunoprecipitated complexes were incubated with 40 μg of bacterially expressed glutathione-S-transferase (GST)-PAK-CD fusion protein prebound to glutathione agarose beads for 30 min at 4 °C. The beads were pelleted, washed with 500 μL of Rac1 lysis buffer, mixed with 1× SDS sample buffer (50 mM Tris-HCl (pH 6.8), 2% SDS, 0.1% bromophenol blue, 10% glycerol, and 100 mM dithiothreitol) and incubated for 5 min at 100 °C. The samples were then separated by 10% SDS-PAGE and immunoblotted with an antibody against Rac1 [60].

### 4.11. Measurement of Intracellular Glucose

The intracellular glucose of NPC cells was measured using glucose assay kit (Abcam, Cambridge, MA, USA) according to the manufacturer’s instructions. Briefly, cells were plated in 24-well plates at the density of 2 × 10^4^ cells per well to allow for attachment overnight. The cells were grown to ≈60% confluence and treated with vehicle or GA for the indicated periods. At the end of the incubation, cell lysates of 1 μL were added to 96-well plates and the volume was adjusted to 50 μL/well with Lactate Assay Buffer before addition of 50 μL Glucose Reaction Mix (composed of 46 μL Glucose Assay Buffer, 1 μL Glucose Enzyme Mix, and 1 μL Glucose Probe) to each well and incubation at room temperature in the dark for 10 min. The absorbance was determined in a microplate reader (EL340 Bio-TEK Instruments, Wihnooski, VT, USA) at 570 nm. Glucose concentration was derived from absorbance using a standard curve.

### 4.12. Determination of GCS Activity

This assay was performed as previously described [27]. A total of 2 μM of NBD-C6-Ceramide was added to the treated cells and then incubated at 37 °C for 30 min. Cells were then harvested by centrifugation at 1000× *g* for 5 min in a microcentrifuge at 4 °C, cell pellets were washed by adding ice-cold PBS. A total of 666 μL of methanol was added to the cells and vortexed for 30 s twice, and then kept on ice for 10 min. In total, 333 μL of chloroform and 333 μL of water was added to tubes, and were further vortexed two times for 30 s then spun at 3000 rpm for 5 min at room temperature. The lipid-containing lower phase was transferred to a new set of glass tubes and lipids were dried down by nitrogen gas. Lipids were then resuspended in 30 μL of chloroform/methanol (2:1, *v*/*v*) and loaded onto high-performance-thin-layer chromatography (HP-TLC) plates for separation. NBD-Glycosphingolipids were used as standards. NBD-GlcCer were normalized to total (NBD-Ceramide + NBD-glucosylceramide), and then normalized to total cell number and time.

### 4.13. Determination of Cholesterol, Sphingolipid, and Ceramide

A total of 50 μL of cell lysates or caveolin-1 and CD55-riched DRM fractions were extracted with 200 μL chloroform plus 200 μL methanol and then subjected to centrifugation at 12,000 rpm for 5 min. The bottom layer was collected and then evaporated under vacuum to a small pellet. The pellet was dissolved in 50 μL ethanol. Cholesterol levels were determined using an Amplex Red Cholesterol Assay Kit according to the manufacturer’s protocol. The amount of sphingolipid and ceramide was quantified by TLC as described by Dobrowsky et al. [61].

### 4.14. Measurements of MMP-2 and -9 Promoter Activities

Both assays were performed as previously described [60]. Briefly, the pGL3-basic (vector), pGL3-control (control), or MMP-2 promoter plasmids were co-transfected with a β-galactosidase expression vector (pCH110) (10:1) into cells using Lipofectamine 2000 following the manufacturer’s protocols (Invitrogen Life Technology, Carlsbad, CA, USA). After 12 h of post transfection, the cells were treated with vehicle, GA, or inhibitor for additional 36 h. For the overexpression assays, cells were transfected with MMP-2 or MMP-9 promoter plasmid and plasmid expressing NF-κB p65 using Lipofectamine 2000. The β-galactosidase expression vector pCH110 was included as an internal control. After 12 h of transfection, cells were treated with vehicle or GA for additional 36 h. The cell lysates were harvested, and the protein expression and luciferase activities were assessed as previously described [60].

### 4.15. In Vitro Invasion Assay

The in vitro invasion assay was performed in triplicate as previously described using transwell chamber units with 8 μm pore polycarbonate membranes [60]. Briefly, approximately 1 × 10^4^ cells were resuspended in 200 μL of serum-free DMEM, seeded into the upper chambers of the Matrigel-coated transwell plates and treated with vehicle or GA. Then, 300 μL of the same medium containing 5% FBS was placed in the lower chamber. After 24 or 48 h incubation periods, cells on the upper surface of the transwell membranes were removed by wiping with a cotton swab, and the cells that had migrated to the lower side of the membrane were stained with a 2% crystal violet solution. For each treatment, 10 randomized fields were counted using a light microscope at 200× magnification. The number of invading cells in each experiment was normalized using the MTT assay (see below) to correct for the effects on cellular proliferation of treatment with vehicle or GA (normalized invading cell number = counted invading cell number/value of OD_570_).

### 4.16. Gelatin Zymography

The culture medium of the treated cells was mixed with 2 × SDS sample buffer without dithiothreitol, incubated for 20 min at 37 °C to denature the MMPs, and to dissociate any complexes of metalloproteinase inhibitors. The samples were separated by 10% SDS-PAGE containing 0.1% gelatin type B. After electrophoresis, the gel was rinsed in 2.5% Triton-X 100 for 1 h at RT and then for 24 h at 37 °C in buffer containing 50 mM Tris-HCl (pH 7.6), 150 mM NaCl, 10 mM CaCl_2_, and 0.02% NaN_3_. The MMPs were identified following staining (0.1% Coomassie blue R250 dissolved in 40% methanol and 10% acetic acid) and destaining (40% methanol and 10% acetic acid) of the gel. Gelatinolytic activities were visualized as a clear band against a dark background of stained gelatin.

### 4.17. Plasmid, siRNA, and shRNA Transfection

Cells (at 70% confluency in a 12-well plate) were transfected with Myc epitope-tagged constitutively active (CA) Rac1, HA epitope-tagged GLUT-1, NF-κB p65 expression plasmids, or with CK2α shRNA MMP-2 or MMP-9 siRNAs using Lipofectamine 2000. The expression of Myc-CA Rac1, HA-GLUT-1, NF-κB p65, CK2α, MMP-2, or MMP-9 in the transfected cells was assessed by Western blot using specific antibodies against HA, Myc, NF-κB p65, CK2α, MMP-2, or MMP-9.

### 4.18. NF-κB Promoter Activity

The assay was performed as previously described [60]. Briefly, pTAI-SEAP, pNF-κB-SEAP, or pSEAP2 plasmids were co-transfected into cells with an EGFP expression vector (at a 10:1 ratio). After 8 h of post-transfection, cells were incubated with medium containing vehicle (−), GA (20 μM), NSC23766 (60 μM), TPCK (10 μM), or PDTC (10 μM) for the indicated periods. For the overexpression assay, overnight cultured cells were co-transfected with the pNF-κB-SEAP and EGFP expression vectors and an NF-κB p65 expression plasmid (10:10:1) for 24 h. The SEAP activity in the medium was evaluated as previously described [60].

### 4.19. Statistical Analysis

Statistical calculations of the data were performed using the unpaired Student’s *t*-test and one-way ANOVA. *p* < 0.05 was considered to be statistically significant.

## 5. Conclusions

This is the first finding that GA activates c-Src to initiate formation of inactive Rac-1-p-CK2α (Tyr 255) complexes by inducing phosphorylation of CK2α tyrosine residue 255, leading to the inhibition of Rac1 activation and blocking the recruitment of Akt into the lipid raft membranes, thereby attenuating the Akt-regulated GLUT-1-mediated sphingolipid synthesis. The declined sphingolipids cause an elevated ceramide level and induce formation of p110α-free p85α-unphosphorylated PTEN complexes in the lipid raft membranes impairing the compartmental regulation of PI3K molecules, accompanied by attenuation of NF-κB regulated MMP-2/-9-mediated cellular invasion. Therefore, the underlying molecular mechanism of GA-induced impairment of the lipid homeostasis of lipid raft membranes affects the compartmental localization of signal effector proteins in the lipid raft membranes. It may reveal a strategy for the theoretical basis of new concepts in anti-cancer invasion.

## Abbreviations

ASM	Acid sphingomyelinase
CK2	Casein kinase 2
GLUT-1	Glucose transporter-1
MMP-2	Matrix metalloproteinase-2
MMP-2	Matrix metalloproteinase-9
NF-κB	Nuclear factor-kappa B
PTEN	Phosphatase and tensin homolog deleted from chromosome 10
PI3K	Phosphatidylinositol 3-kinase
PIP_2_	Phosphatidylinositol-4,5-bisphosphate
PIP_3_	Phosphatidylinositol-3,4,5-trisphosphate
Akt	Protein kinase B
Rac1	Ras-related C3 botulinum toxin substrate 1
shRNA	Short hairpin RNA
SP-1	Stimulatory protein-1
TSC	Tongue squamous carcinoma
